# Detecting small and cryptic animals by combining thermography and a wildlife detection dog

**DOI:** 10.1038/s41598-020-61594-y

**Published:** 2020-03-23

**Authors:** Denise Karp

**Affiliations:** University of Zurich, Department of Evolutionary Biology and Environmental Studies, Winterthurerstrasse 190, 8057 Zurich, Switzerland

**Keywords:** Behavioural ecology, Biodiversity, Conservation biology, Evolutionary ecology, Animal behaviour

## Abstract

Small and cryptic species are challenging to detect and study in their natural habitat. Many of these species are of conservation concern, and conservation efforts may be hampered by the lack of basic information on their ecological needs. Brown hare (*Lepus europaeus*) leverets - one example of such a small, cryptic and endangered animal - are notoriously difficult to detect, and therefore data on wild leverets are virtually non-existent. Novel technologies and methods such as thermal imaging and the use of wildlife detection dogs represent suitable means for the detection of such species by overcoming the problem of camouflage, using heat or scent emission respectively. Our study on brown hare leverets provides information on how to apply these new techniques successfully for the detection of small and cryptic species, thus enabling the collection of data that was previously inaccessible (e.g. behavioural observation, radio tagging). We found that the choice of method should be made according to vegetative structure. While the handheld thermal imaging camera is best used in areas with no or low vegetative cover, the thermal drone can be used up to medium vegetative cover, whereas the detection dog method is best applied where vegetation is very dense and not suitable to be searched using thermography. Being able to search all sort of different vegetation types, our combined approach enables the collection of a balanced and unbiased dataset regarding habitat type and hence selection of study specimen. We hope that the use of these new techniques will encourage research on many cryptic species that formerly have been neglected because they could not be detected using conventional methodologies.

## Introduction

Many animal species show a form of crypsis – the ability to minimize detection by other animals – either to avoid being predated or to avoid detection by potential prey^1^. The simplest form of crypsis is background colour matching which is of relevance to visually oriented animals including humans^2^. Predator avoidance is especially important for dependent offspring, and accordingly, the young of many species are exceptionally well camouflaged^3^. Due to their cryptic and elusive nature, these highly camouflaged animals can represent a considerable challenge for researchers to detect and study^4^. Many of these cryptic species are classified as being threatened and conservation efforts often are restrained by the lack of basic information on their ecological needs^5,6^. While for some study objectives (e.g. density estimation or diet composition) indirect methods including scat collection, camera or hair trapping are fully adequate^7–9^, for other studies there is an urgent need to find the animal itself. For example, if behavioural data needs to be collected or if animals need to be fitted with radio- or GPS-collars. Furthermore, our knowledge of a species is often restricted to the behaviour displayed during daytime. In contrast, activities at night are often poorly known^10,11^. Therefore, methods are needed to facilitate the collection of data throughout the complete life cycle and all activity phases of a given species. Only then can adequate species management or conservation measures be implemented.

Thermal imaging cameras use emitted heat (infrared radiation) instead of visible light to create an image^12^. As thermal cameras require thermal but not visual contrast, they provide accurate vision even when camouflage or darkness render normal eyesight useless^12^. Thermal imaging techniques to study wildlife were first tested in the late 1960’s on white-tailed deer, *Odocoileus virginianus*^13^, and have afterwards been used to detect, survey or observe a range of different mammals, birds and invertebrates^12^. To date, the smallest mammals that have been systematically studied using thermal imaging are ungulate fawns, adult hares (*Lepus europaeus*), adult rabbits (*Oryctolagus cuniculus*) and hedgehogs (*Erinaceus europaeus*)^14–17^. However, some studies have reported a rather low detection rate, stressing the fact that alternative approaches are needed^15,18^.

With their superior sense of smell, wildlife detection dogs represent an alternative method to search for cryptic wildlife or their remains. Wildlife detection dogs have recently been used to detect remains (mostly scats) of various different animals including grizzly bear (*Ursus arctos*^19^), barred owl (*Strix varia*^20^), koala (*Phascolarctos cinereus*^21^) and many others, but less so to detect target animals themselves (but see^22^: desert tortoise (*Gopherus agassizii*)^23^: pygmy bluetongue lizard (*Tiliqua adelaidensis*)^24^: kiwi (*Apteryx spp*.) and^25^: franklin’s ground squirrel (*Poliocitellus franklinii*)). For live target detection, a wildlife detection dog must find the target species, indicate its find by showing a trained alert behaviour and do both of these tasks without harming the target animal, itself, its handler or any other human or wildlife^22^.

Using the brown hare leveret (*Lepus europaeus*) as a representative for small and cryptic animals, we describe the pros and cons of using thermography (handheld and airborne) and a wildlife detection dog to detect cryptic wildlife. Their lack of motion during most of the day, small size, and cryptic fur coat make leverets visually merge with the background. Traditional methods such as spotlighting or line transect counts hence are not suitable for their detection. Furthermore, capture methods such as box-trapping are not successful in trapping leverets younger than one month^26^. Finally, indirect detection of young through adult individuals or searching for nests is nearly impossible because mothers interact with their young only once a day for less than five minutes^27^ and do not provide any protective cover, presumably in order to reduce the probability for predators to detect cues about the location of their young. Lacking a method to systematically detect leverets, researchers have so far been highly constrained in their ability to study the early life-stage of the brown hare. However, adequate management of a species requires knowledge of its complete life cycle. This is particularly the case for hares, where increased postnatal mortality has been identified as an important factor explaining the decline of brown hare populations in Europe^28,29^.

Here we provide information on the advantages and drawbacks of three methods useful to detect cryptic wildlife: (i) handheld thermal imaging, (ii) airborne thermal imaging and (iii) wildlife detection dogs. We also provide application-oriented information on each method and a qualitative evaluation of detection efficiency.

## Methods

We searched for leverets in two different study sites in the North-Western part of Switzerland. Study site “Reinach” (47°28′47.760″N 7°35′03.879″E) was located close to the village of Reinach at an elevation of 310 masl, was 1.01 km^2^ in size, and included 10 km of navigable roads. The second study site “Selzach” (47°11′48.615″N 7°27′59.658″E) was located near the village of Selzach, at an elevation of 430 masl, was 3.1 km^2^ in size, and had 26 km of navigable roads. Both study sites were characterized by mixed agricultural landscape with wheat, grassland, sugar beet and corn as main crops. Agricultural usage included conventionally used farmland as well as ecological compensation areas and differed only little between the two study sites (Supplementary Material Fig. S1). Adult hare population density in Reinach was more or less stable around 3.5 hares/km^2^ over the three study years, while in Selzach, population density increased from 3.6 hares/km^2^ in 2013 to 11.4 hares/km^2^ in 2015 (official yearly spotlight counts^30^).

We searched for leverets during 26 months of the years 2013 (mid-February–start October), 2014 (start February–end October) and 2015 (mid-January–end October). Those leverets we were able to capture were measured in order to determine their age^31^. All methods were approved, and the study-permit was issued by the Economic and Health Affairs Department of the canton of Basel-Landschaft, Switzerland (permit Nr. BL443, valid for both study sites). All methods were performed in accordance with the relevant guidelines and regulations. All statistical analyses were carried out using the software R^32,33^.

### Thermal imaging: handheld

For the handheld thermal imaging method, we used a FLIR Scout TS-32r Pro (FLIR systems, Inc., USA) thermal imaging camera with a 65 mm lens (detailed information on the camera is given in the supplementary material A). We searched agricultural fields of different usage until vegetation succession - in terms of height and density - did not allow for target detection anymore. The current vegetation height and density on each field was used to determine the area searched. The more vegetation is growing on a field the more the sight is restricted leading to less area being searched. For fields with no vegetation (e.g. acre) the whole field was assumed to be searched. Of course, this only applies to flat landscape with no sight obstructing structures. After determining the area searched for each field the numbers were summed up to get the total area searched.

**Figure 1:**
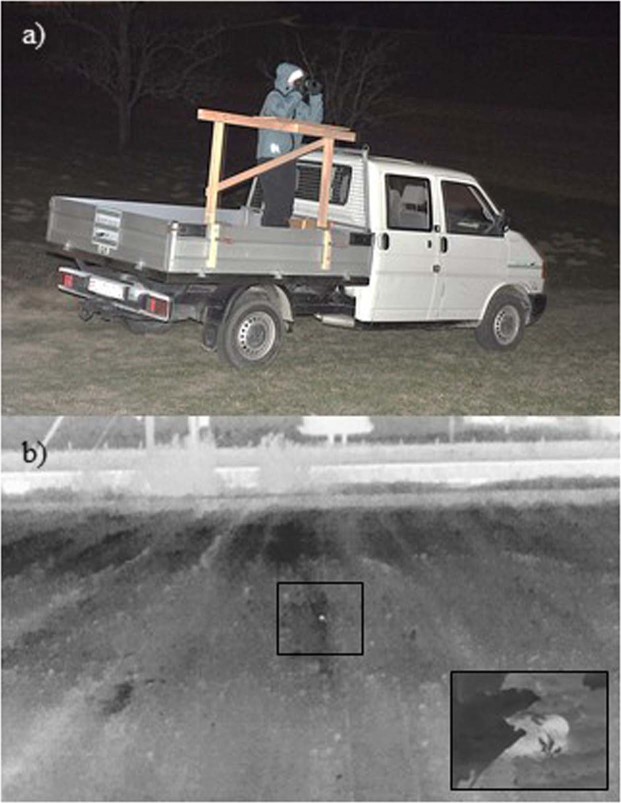
(**a**) Setup for the application of the handheld thermal camera. (**b**) Suspicious thermal signature in a distance of about 40 meters, corresponding to a leveret in terms of size, shape and brightness. Identification is not possible, thus close inspection is necessary. Small picture: close-up of a thermal signature of a leveret from a distance of 3 meters. Both pictures have been captured with a handheld FLIR Scout TS-32r Pro thermal camera.

The application of the handheld thermal camera required one “viewer” standing on the back of a pick-up truck and a “driver” manoeuvring the truck. Driving speed was constantly adjusted depending on type, height and density of vegetation being searched: fast (15 km/hr) for bare ground with no vegetation (acre) and slower (5–10 km/hr) for more vegetated areas. During searching, the viewer had her elbows positioned on a platform fixed to the pick-up while constantly looking through the camera (Fig. 1a). The platform enabled stabilization of the viewer itself and thus a stable handling of the camera. While driving along the field paths, the angle of the camera was always orthogonal to the searched agricultural field, resulting in an optimal view along the seed rows. While keeping the horizontal angle constant, the viewer continuously panned the camera up and down (vertically) to capture thermal signatures as close and as far as possible from the car. We also used the handheld thermal camera for on foot searches of areas not accessible by car. On foot searches represent an opportunistic search method as the camera handling while walking does not allow for systematic detection. Hence on foot searches are particularly useful for small-scale habitat. In addition, we used the handheld thermal cameras for non-invasive behavioural observations in complete darkness (e.g. suckling behaviour of hares, see supplementary material B).

Thermal imaging does not allow for species identification, unless size, shape or behaviour of the target species are very distinctive and not to be confused with any other species present in the area^12,34^. For most suspicious thermal signatures (handheld and airborne method), approaching and illuminating was required for identification (leveret vs. e.g. resting birds or earth clumps) (Fig. 1b).

### Thermal imaging: airborne

With the airborne method we were able to search areas otherwise obstructed by vegetation when using the ground-based view of a handheld camera. For the airborne method we used two different unmanned aerial vehicle (UAV) systems: microdrones md4–200 (Siegen, Germany) and AEROdron UAV (Zug, Switzerland), both consisting of a vertical take-off and landing (VTOL) quadrocopter with its corresponding ground station system (specifications are given in the supplementary material C).

**Figure 2:**
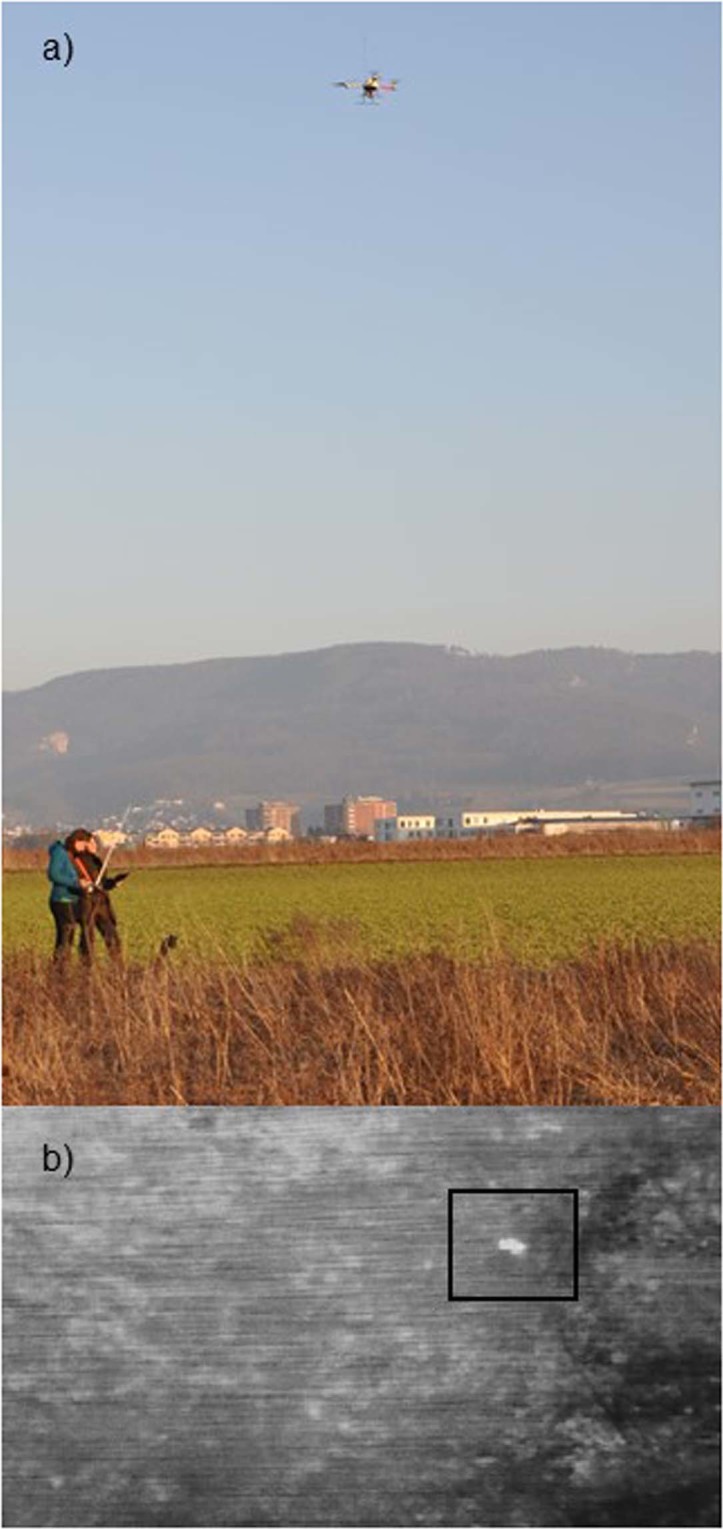
(**a**) Using a thermal drone to search for cryptic wildlife. (**b**) Thermal picture taken with a FLIR Photon 320 mounted on a microdrones md4–200 quadrocopter nine meters above ground level. The framed spot represents a leveret located within medium - high vegetation density (fallow land).

Searching for leverets involved the manual control of the thermal drone along systematic transects and the continuous monitoring of the screen displaying the thermal images, until a suspicious thermal signature was detected (Fig. 2a,b). In that case, the thermal drone was put into a stabilizing mode that allowed the aircraft to maintain a level flight without further input from the pilot. After checking the thermal signature, the pilot continued to fly the drone along the transect. We decided for manual flights instead of autopilot, enabling us to continuously modify flight speed according to vegetation and wind characteristics. Furthermore, throughout the flights, frequent stops were necessary, to watch suspicious thermal signatures and assess whether to inspect these or not. For instance, fast moving signatures could be classified as mice, making an approach unnecessary. Flight height was kept constant at 9 meters above ground level and average speed was approximately 1.5 m/s to be able to detect very small thermal signatures characteristic of leverets. Search bout length was limited by battery power and the area being searched was determined by the transect length.

### Wildlife detection dog

**Figure 3:**
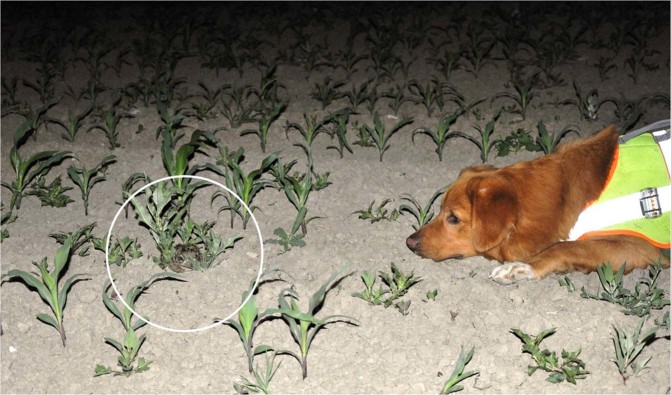
Wildlife detection dog performing its trained alert upon detection of a leveret: laying down with its head on the ground and the snout pointing into the direction of the target.

One male dog was trained to search for, and upon encounter alert on, leverets (for details on the selection process and training procedures see supplementary material D). The alert behaviour – a specific behaviour that the dog performs immediately and unprompted upon finding the target^22^ – was defined as lying down and pointing with the head in the direction of the leveret, leaving a space of about 30–50 cm between dog and leveret (Fig. 3). During searching the dog roamed free (distance to handler 1–30 m) and the handler only interfered when the dog obviously missed a part of the search area. The search area was defined by the handler and continuously adapted to the dog’s mental and physical performance as well as wind and general weather conditions. While individual search bout length, i.e. the area being searched, was restricted by the dog’s mental/physical performance, after appropriate recreation the detection dog was able to perform during multiple search bouts per session. The dog never performed the alert behaviour when there was no leveret (false positive). The dog also never chased after a moving/bolting leveret but always remained in its alert position.

### Detection probability and efficiency

We compared average vegetation height at the detection locations for the two thermal imaging devices (handheld and airborne) using a Student’s *t*-Test (function: t.test^32,33^). Such data was available for 50 individuals (45 for the handheld camera and five for the thermal drone). Furthermore, for the handheld thermal imaging method, we estimated detection probability (P_H_). P_H_ was determined using leverets that had been tagged before, so we knew the leverets’ rough location. Without knowing the exact location, we then searched the fields in the perimeter of roughly 100 ×100 meters and recorded whether we could detect the animal. Searching was done in exactly the same way as in the usual procedure described above (section “Thermal Imaging: Handheld”). Afterwards we checked whether the exact location of the leveret lied within the searched area (in case we could not detect the leveret using the handheld thermal camera). We tested the effect of the leveret’s age [days], the time after sunset [min.] and the average vegetation height [cm] on the probability to detect leverets with the handheld thermal camera by fitting a generalized linear mixed effects model with litter ID as random factor (function: glmer, family binomial, package: lme4, r-project^32,33^). We used the leveret’s age as a proxy for its size as these variables are well correlated and we expected easier detection of bigger leverets. The variable time after sunset was used as a proxy for the activity level of the leverets, which decreases over the course of the night, and we expected inactive leverets to be harder to detect (time of data collection in minutes after sunset: Ø ± s.d. = 139 ± 81 min., range = 15–346 min.). As vegetation conceals the leveret’s thermal signature, we expected vegetation height to negatively influence detection probability.

### Ethical approval

All applicable international, national, and/or institutional guidelines for the care and use of animals were followed (permit Nr. BL443).

## Results

65 individual leverets from 41 different litters were detected, caught and radio tagged. 14 additional leverets (from 9 additional litters) were detected but not caught as these individuals were either too old and hence escaped or we failed to catch them upon first detection and could not detect them anymore. Captured leverets were between 1 and 22 (mean ± s.d. = 8 ± 6.2) days old upon first detection. We could not determine the age of the non-captured leverets. Body weight at capture ranged from 133–799 g (mean ± s.d. = 340 ± 187 g). For details on the number of leverets found, area coverage and time investment for each method see Table 1. In total, we spent 895 hours (85% handheld thermal camera, 12% thermal drone, 3% wildlife detection dog) searching for leverets using the three different methods described above. Average time investment to find any leveret was 12.3 hours (including all three methods). However, as the detection of littermates was not independent, the average time investment to find leverets from different litters is more relevant and was found to be 17.2 hours (including all three methods). Our relatively low success rate was likely influenced by both, low detection probability and low brown hare densities at the study sites.

**Table 1:** Details on detection success, search time (hr) and searched area (ha) for the three different detection methods used to find leverets.

	*n* leverets detected (*n* different litters)	Ø area searched/bout [ha ± s.d.]	maximum area searched during one bout [ha]	area searched [ha]^a^	Ø search bout length [min. ± s.d.]	total time spent searching [hr]	time searched/litter [hr]^b^	Ø area coverage [ha/hr]
Handheld thermal imaging camera	63 (46)	70 ± 43	175	6700 (17^1^, 281^2^)	180 ± 62	759 (85%)	16.5	23.8
Thermal drone	7 (4)	0.58 ± 0.45	2.7	54	66 ± 30	106 (12%)	26.5	0.5
Detection dog	3 (2)	0.23 ± 0.13	0.8	13 (1^1^, 22^2^)	23 ± 8	30 (3%)	15.0	0.6
Chance find	6 (4)							

^a^For the handheld thermal camera and the detection dog, we only recorded the area searched per search bout during the last field season (2015). Therefore, the area being searched corresponds only to a fraction of the total number of litters^1^ and the total time investment^2^, both indicated in brackets.

^b^As the detection of littermates is not independent, we provide data on the detection of the number of different litters but not of individual leverets.

Detection distance (distance from viewer to target) applying the handheld thermal camera ranged from 16 to 190 m (mean ± s.d. = 61 ± 33 m). Because of the restricted angle of the camera close to the viewer’s body, the area <10 m to the observer was considered as not being searched. Table 2 lists species other than hares detected using the handheld thermal camera. Average vegetation height at the detection location was ~70% shorter for the handheld thermal camera (mean ± s.d. = 11 ± 9 cm) compared to the thermal drone (mean ± s.d. = 38 ± 7 cm, *t* = −7.357, *P* < 0.001), while maximum vegetation height was 30 cm for the handheld method and 46 cm for the thermal drone respectively.

**Table 2:** List of species (ordered by size) detected using the handheld thermal camera during the leveret surveys. All observations were made after sunset in complete darkness. The list is not complete, as not all detected animals could be identified to species level (e.g. Muridae spp. or Arvicolinae spp).

Mammals	Birds
Roe deer (*Capreolus capreolus*)	Stork (*Ciconia ciconia*)
Wild boar (*Sus scrofa*)	Barn owl (*Tyto alba*)
Badger (*Meles meles*)	Long-eared owl (*Asio otus*)
Stone marten (*Martes foina*)	Mallard (*Anas platyrhynchos*)
Fox (adult and pup) (*Vulpes vulpes*)	Pigeon (*Columba livia domestica*)
Domestic cat (*Felis silvestris catus*)	Common snipe (*Gallinago gallinago*)
Adult hare (*Lepus europaeus*)	Jack snipe (*Lymnocryptes minimus*)
Hedgehog (*Erinaceus europaeus*)	Barn swallow (*Hirundo rustica*)
	Bluethroat (*Luscinia svecica*)
	Skylark (adult, fledgling and nest) (*Alauda arvensis*)

Out of 109 search attempts (determination of P_H_), we were able to detect the target leveret in 39 cases, resulting in a detection probability (P_H_) of 36% for the handheld method. P_H_ was positively influenced by the leveret’s age as a proxy for its size (model estimate = 1.77, SE = 0.87, χ^2^ = 4.57, *P* = 0.033) but negatively by average vegetation height (model estimate = −3.09, SE = 2.16, χ^2^ = 9.39, *P* = 0.002) and time after sunset (model estimate = −1.68, SE = 0.73, χ^2^ = 8.96, *P* = 0.003).

## Discussion

The detection of small and cryptic species in their natural habitat poses a significant challenge for researchers. As our study on brown hare leverets shows, thermal imaging and wildlife detection dogs represent suitable methods for the detection of such species. With a birthweight of 100–150 g, brown hare leverets are the smallest mammal being searched for systematically using thermal imaging so far^35^. Moreover, it is the first time a wildlife detection dog has successfully been trained to detect live leverets – a target difficult to detect olfactorily^36^ and very likely to elicit chasing behaviour once bolting. Furthermore, the combination of thermography and a wildlife detection dog has never been done before and is in that sense unique. This combined approach allows to cover all sort of vegetation structures (open, sparse and closed) and hence collect an unbiased/balanced dataset in terms of vegetative cover or habitat type respectively. The application of the methods presented here – whether individually or in combination – hugely increases the possibilities for collecting data, and ultimately for gaining a much-needed understanding on the ecology and behaviour of small and cryptic species at all life history stages. This may include research on many endangered farmland species such as Eurasian skylarks (*Alauda arvensis)*, grey partridges (*Perdix perdix*), Northern lapwings (*Vanellus vanellus*) or European hamsters (*Cricetus cricetus*), but also, on species outside the farmland context such as for example sand cats (*Felis margarita*) in the desert, bird nests on cliffs, or hedgehogs (*Erinaceus roumanicus*) in the park. Another field of application is the detection and elimination of invasive species^17,37,38^.

### Thermal imaging: general

**Figure 4:**
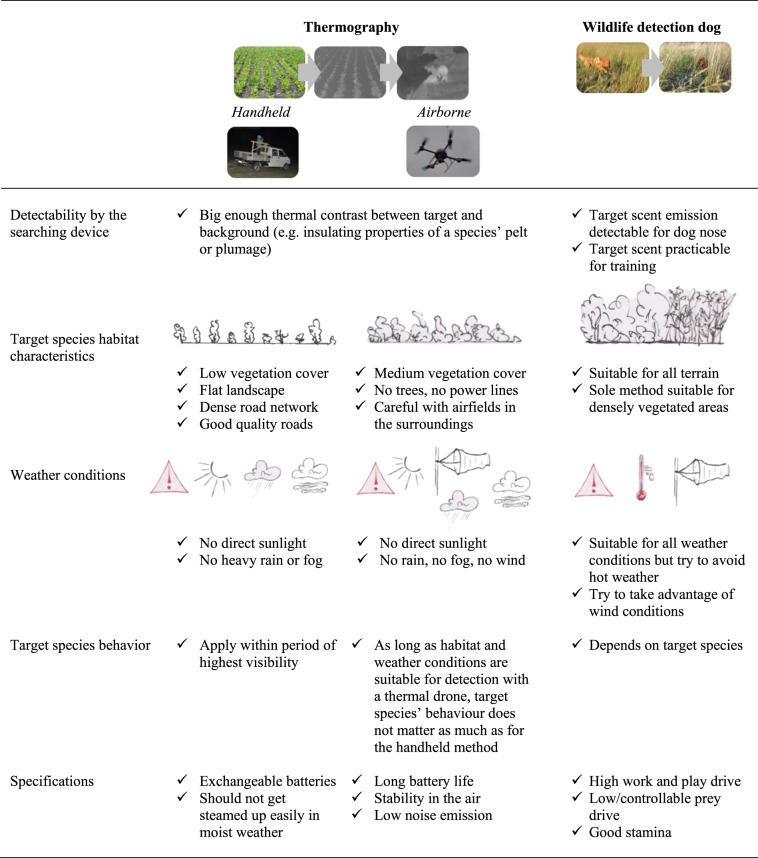
Summary of key factors to consider for successful cryptic wildlife detection.

While the acquisition of a specific search image for the application of the thermographic methods is fast and simple for areas with no vegetation, it requires a lot of practical experience when thermal signatures are small and indistinct i.e. in vegetated areas. Besides considerable search experience some additional factors must be considered for successful wildlife detection using thermal imaging: (i) the characteristics of the target species’ habitat; (ii) the thermal contrast between the target and the background; (iii) weather conditions and (iv) the target species’ behaviour (Fig. 4).

(i) When using thermography, an unobstructed view to the target is the most important factor to account for. While displaying emitted heat, thermal imaging devices are not able to detect targets behind objects that cannot be penetrated by heat^12^. Therefore, the main problem with the detection of small animals when using thermography is the obstruction of the target by vegetation^11,13,39,40^. Depending on the size of the target as well as vegetation height and density, only very small parts of the animal or nothing at all is visible through the thermal camera. Thus, by reducing driving or flying speed respectively with increasing vegetation density and/or height, the detection of small signatures can be improved. For the handheld method, but not for an aerial view with the drone, one other aspects of the target species’ habitat can affect the detection rate: the relief. Depressions and elevations (or rocks - larger than the target) in the terrain can lead to undetected targets, due to the flat angle when using the handheld method.

As vegetation normally grows vertically (straight upwards), obstruction of the target by vegetation is maximized when viewing from the ground horizontally into the vegetation layer. We found that with the handheld camera, the probability to detect a leveret was significantly lower with increasing vegetation height. Hence, the handheld method is only suitable for habitats with no or low vegetation cover. However, shifting the ground view from the handheld camera into an aerial view with the drone enabled target detection up to much higher vegetation density and height (70% taller in our study). As soon as vegetation grows very dense or horizontally - branching out to the side rather than growing straight upwards (e.g. full-grown sugar beet or forest canopy) - thermography reaches its application limit (in our study vegetation height> 46 cm) even with an aerial view.

(ii) For successful detection using thermal imaging, sufficient thermal contrast between target and background is required^12,41^. Thermal contrast is increased when inanimate objects (background) have low temperatures compared to the target animal, making thermal imaging most efficient in cold environments^14,15,42^. In contrast, direct sunlight hampers detection of target animals as inanimate objects heat up, resulting in false positive detections^14,15,38,42^. Consequently, searching during daytime requires overcast weather for the thermal devices. We found that as soon as the sun set (no direct sunlight), thermal contrast was large enough for detection. This was still the case in midsummer, when ambient temperature remains high after sunset. Generally, animals with well insulating plumage or fur coats will be less visible by thermal imaging compared to animals that lose more heat to the surrounding environment (e.g. when they are wet)^12^. Similarly, ectothermic animals are difficult to detect using thermography due to the small thermal contrast between target and background^43,44^.

(iii) Some weather conditions such as water vapor present in the air lead to the absorption of infrared radiation resulting in blurry images and the diminishment of the thermal signature^12,42,45^. Hence, high air humidity, dense fog or heavy rain, reduce detection probability and render thermal imaging unsuitable^12,42,45,46^. As the technical parts of a thermal drone usually are humidity sensitive and wind speeds of more than 5 km/hr result in the drone drifting away from its original transect and cause shaky images, making it impossible to systematically search an area and detect suspicious thermal signatures on screen, the thermal drone should only be used under conditions with no rain, no fog and no wind.

(iv) Considering the target species’ behaviour when using thermography, implies focusing on the time of day when targets are most visible. Depending on target species, this can apply either to the active or inactive period. Regarding our study species, leverets are most visible when they are active. Activity peak falls between 60 and 100 (range 30–200) minutes after sunset when leverets leave their daytime hiding spot and move towards their birth place where they meet their littermates and will be suckled by their mother^47^. After suckling, littermates spread out and hide until the next suckling event on the following day^27^. Apart from suckling, leverets spend most of their time immobile, lying down motionless to be as inconspicuous as possible. This specific daily activity pattern explains the negative effect of “time after sunset” on detection probability. In contrast to leverets, a specific target species may be most visible when it is inactive resting in low cover habitat for example. We conclude that one should account for the target species’ daily activity pattern when deciding on the best time of day to apply thermal imaging methods.

### Thermal imaging: handheld

If the above-mentioned (“Thermal Imaging: General”) aspects are respected, the handheld thermal camera model used for this study overall was a very efficient and easy-to-use tool that was technically reliable, featuring a waterproof and rugged design, requiring only a set of charged batteries and a pick-up truck. However, the handheld method requires a team of two researchers (driver and viewer). Compared to airborne thermal imaging systems and detection dogs, the handheld method covers a much larger area during a specific time period (Table 1). However, at the same time this method is maximally restricted by vegetation compared to the other two methods described here and can thus only be used for sparsely vegetated areas.

### Thermal imaging: airborne

While the two aerial systems we used for our study were not yet technically optimized (poor battery life and technical reliability), rapid improvements and widespread use of drone technology in the meantime caused the development of low-cost aerial systems with longer battery performance, improved technical reliability and better stability in the air^46^. We thus expect future applications to be more efficient compared to our study and are thus convinced that thermal drones represent a very promising tool for detecting cryptic wildlife^46,48–51^. In general, thermal drones are relatively easy to handle and require only little training before confident use. When deciding on using an airborne thermal imaging system to effectively detect cryptic animals or generally to be used as a management or research tool, the following features should be met: maximized flight time, a steady flight pattern, robustness against wind, ideally rainproof, minimal noise emission, technical reliability as well as a compact design (most important: screen attached to remote control for fully mobile use, enabling operation by a single person).

### Wildlife detection dog

Compared to thermal imaging, a detection dog is not limited as much by weather conditions such as rain, wind, humidity or direct sunlight nor by vegetation or rugged terrain^52–54^. Yet all these external conditions do influence the scent’s behaviour, making it more or less difficult for the dog to detect the target scent particles and can thus affect detection probability^36,55,56^. Furthermore, hot weather will lead to increased panting, resulting in a decreased scenting ability and rapid exhaustion^52,57^.

When using a wildlife detection dog for live target detection care must be taken to select a suitable dog, that is trainable and can be handled (controlled) in immediate proximity to wildlife (low or controllable prey drive). If the study species itself is not required to be found but only evidence of its presence, wildlife detection dogs can be extremely valuable to find scats, hair, burrows, scent markings, food remains, etc. of the study species^58^. Generally, training a detection dog for a specific project requires knowledge on how and where target scent samples can be collected. For live target detection the acquisition of training samples (residual scent in the first place and target animals in a second step) can be a significant challenge because isolating the target’s scent can be difficult and keeping target animals for training purpose is most often linked to ethical concerns.

Similar to thermography, where thermal contrast must be large enough for detection, using dogs to detect wildlife implies that the target is detectable for a dog’s nose. While animals always release hundreds of different odorants, they can use strategies to either limit their release or make their perception difficult^36^. While the latter is merely done by microhabitat choice, the former strategy involves reducing the metabolic rates which reduces the release of metabolic odorants^59–61^. Animals can also reduce the emission of odorants stemming from their surface by minimizing the surface in contact with the air^36,62^. Prey hiding from olfactorily hunting predators can choose areas experiencing updrafts, meaning that the odorants raise up with the air to a level where they can no longer be perceived by the predator^36^. Furthermore, in areas with turbulences, odour plumes meander and odour concentration can vary unpredictably making it hard to detect the odour source^36^. Turbulences and high wind velocity also cause the odorants to disperse more rapidly, inducing a decrease in odour concentration, which shortens the distance over which an olfactory predator can locate its prey^36^. We found that the use of these strategies requires dogs (and predators) to get within a close distance before being able to detect the scent and overall results in a low detection probability^62–65^. Similar to the observations by French & French^63^ of grizzly bears predating on elk calves, we observed our dog having difficulties in locating the source of the scent and sometimes passing the leveret within a meter. We also observed a pet dog (off-leash) and a fox to walk by very close (ca. 3–5 m) to a pair of sibling leverets waiting for their mother to be suckled in a harrowed acre (no vegetation) without noticing them. Based on these observations, we consider a very young (up to one week) leveret’s scent to be detectable only within a very short range (about 20–50 cm upwind), yet older leverets were easier to detect for the dog, likely due to increased odour intensity, confirming findings of Autenrieth & Fichter^62^. Hence, the detection of leverets requires a wildlife detection dog to thoroughly search an area to not miss a present leveret and therefore, the covered area is much smaller compared to a dog searching for targets with a better detectable scent. Although the dog’s area coverage was small, the time needed to find one litter was lowest compared to the two thermal imaging devices. Furthermore, a detection dog is the only method so far able to find leverets in dense vegetation and under unfavourable weather conditions. The use of only one dog in our study is a limitation to the generalisation of our results as performance can vary between dogs and handlers (handling itself but also training methods and skills)^22,66–69^. The decision to only train one dog was an ethical one: the final step within the training of dogs to detect live targets, requires the dog to be trained on the target itself, which puts a lot of stress on the target animal, especially if it is a prey. We were aware of this stress and wanted to limit exposure for the individuals (maximally two exposures per individual). Training a second dog would have doubled the number of individuals affected by this stressor. While we cannot conclude whether detection dogs are generally suitable for detecting wild leverets, there exists extensive literature on successful applications of wildlife detection dogs on a near infinite variation of wildlife related scents^58^.

## Conclusion

When searching for cryptic animals, the choice of method should primarily be based on vegetation characteristics (height and density). The handheld thermal camera is very efficient to search large areas with no or low vegetation cover, in a flat terrain with a dense road network to increase area coverage. Application time using the handheld camera is restricted to overcast days or nights and ideally should overlap the period when the target species is most visible. While the handheld method is restricted by the obstructing effect of vegetation, the thermal drone and the wildlife detection dog complement the detection of cryptic species in more dense vegetation but in contrast can cover a much smaller area per time. The thermal drone is best used in areas with medium to high vegetation cover when there is no rain and wind and during overcast days or at night. Not only does thermal imaging enable detection of cryptic species, it also has great potential to investigate behaviour of nocturnal animals, a task which has often been neglected due to poor visibility^10,11^. Finally, the detection dog is helpful in covering areas not accessible with thermal imaging – areas with very dense vegetation cover or vegetation that grows horizontally (e.g. fully-grown sunflower fields or areas below tree cover) – during any time of day and weather condition. When aiming at detecting a well-balanced set of study animals with regard to habitat type, we recommend applying all of the three methods described in this manuscript. A summary of our recommendations can be found in Fig. 4.

Applying the methods presented here can enable the collection of data that formerly was not accessible and hence improve the full ecological understanding of cryptic species, thereby facilitating adequate management and conservation measures where needed. We hope that the presented methods will help to stimulate much-needed research on cryptic species.

## Supplementary information

Electronic Supplementary Material.

## Data Availability

The datasets generated during and/or analysed during the current study are available from the corresponding author on reasonable request.
