# Supplementary material for: Detecting small and cryptic animals by combining thermography and a wildlife detection dog
Source: Sci Rep. 2020 Mar 23;10:5220. doi: 10.1038/s41598-020-61594-y (PMC7090052; doi:10.1038/s41598-020-61594-y)
Supplement: Supplementary file 1 — Electronic Supplementary Material. [file 41598_2020_61594_MOESM1_ESM.pdf]

Supplementary Material

Scientific Reports

Detecting small and cryptic animals by combining thermography and a wildlife detection dog

DENISE KARP
University of Zurich, Department of Evolutionary Biology and Environmental Studies
denise.karp@ieu.uzh.ch

A: Specifications on the handheld thermal imaging camera FLIR Scout TS-32r Pro

The FLIR Scout TS-32r Pro thermal imaging camera was equipped with a 65 mm lens which collects and focuses radiation in the 7.5 – 13.5-micron spectral region and has a field of view of 7° (H) × 5° (V) when not using the digital zoom. The camera weighed 1.05 kg and had a small 8 x 7 mm monocular viewfinder. The resolution was 320 x 240 pixels and its thermal sensitivity was <50 mK @ f/1.0. The camera was powered using 4 AA NiMH batteries whilst operating time varied according to ambient temperature and age of the battery between one and four hours.

B: Additional application of the hand-held thermal camera

Besides the active searching for leverets, we also used the handheld thermal camera (i) to find siblings of a known leveret or (ii) for behavioral observations. (i) In European hares, siblings from one litter always meet at the same location every dusk for suckling (Karp 2019). We took advantage of this behavior by returning to the detection location around sunset the next day after a leveret had been detected and waited for the siblings to gather at the suckling spot. This made it very easy to capture or count all live leverets from one litter. (ii) Given that vegetation is not obstructing the view, thermal imaging allows for non-invasive behavioral observations in complete darkness. For instance, suckling behavior can be observed with no need of artificial lighting and disturbance of the animals during this sensitive period (Karp 2019).

C: Specifications on the airborne thermal systems

Between May 2013 and June 2015, we used a UAV system developed by microdrones GmbH in Germany. The airborne subsystem involved a md4-200 quadrocopter weighing around 900g, powered by four lithium polymer batteries (2400 mAh 4 cell LiPo) allowing for flights lasting 13 – 21 minutes (mean ± sd = 18:03 ± 01:57) depending on age of the batteries as well as wind conditions and air temperature. Maximum payload of the md4-200 quadrocopter was 200g. The onboard technology consisted of several modules for navigation: Global Positioning System (GPS) receiver, Inertial Measurement Unit (IMU), 2-way radio-controlled data transmission/communication with the UAV (video and telemetry transmitter) and the main controller, an accelerometer–gyro system, and a pressure altimeter. The telemetry range of the quadrocopter was 150 meters. For flight operations during night time we equipped the microdrones md4-200 with a small positioning light (Knog frog strobe LED light) to assess flight direction. For thermal image acquisition a FLIR Photon 320 (FLIR systems, Inc., USA) sensor with a 19 mm lens (total weight 110g) was mounted onto the quadrocopter, turning it into a thermal drone. This was achieved using a gimbal system that ensured continuous downward-looking of the camera, stabilization of the video images and equilibration of the vibrations during the flight. The attached sensor was an uncooled long wave thermal imager. The ground station subsystem consisted of the ground station software (md-cockpit, microdrones GmbH) installed on a tablet-PC, the datalink (frequency 2.4 GHz), a video receiver and a remote control for flight operations. The software provided real-time monitoring of flight data, such as height, distance, battery power and GPS connectivity as well as the picture of the thermal sensor for on

spot evaluation of the image. The ground station subsystem was integrated in a 5.5 kg pelicase box (PELI^{TM} 1660, 80x58x49 cm (LxWxH)) which could be carried to the specific operation area.

From August to October 2015 we used a different UAV system built by AEROdron GmbH (Zug, Switzerland). The quadrocopter weighed 1.7 kg and was powered by 2 lithium polymer batteries (RedChunk 3300 mAh 4S 35-70C LiPo) allowing for flights lasting 9 – 22.5 minutes (mean ± sd = 16:14 ± 04:02) depending on age of the batteries as well as wind conditions and air temperature. The quadrocopter was equipped with a FLIR QUARK 640, an uncooled long wave thermal sensor with a 19 mm lens weighing 24 grams. The ground station subsystem of the second UAV system was very compact, consisting only of the remote control for manual control of the quadrocopter and a small 7-inch display for real-time monitoring of the thermal images.

Checking on suspicious thermal signatures differed for the two different UAV-systems used. Because the ground station with the screen displaying the thermal images was not mobile for the microdrones system, we needed two people to confirm suspicious thermal signatures: one person monitored the screen which guided a second person who walked towards the thermal signature, using walkie-talkies (Switel WTC 600, Granges-Paccot, Switzerland). The compact and portable design (screen attached to remote control) of the AEROdron system allowed for direct feedback when inspecting suspicious thermal signatures without the need of a second person and guidance via walkie-talkie. As a disadvantage, the noise level of the UAV built by AEROdrone was much higher compared to the md4-200 quadrocopter. However, we did not observe any difference in the leveret’s behavior using either system.

D: Wildlife detection dog training procedure

We selected the breed “Nova Scotia Duck Tolling Retriever” as it brings along all the desired traits characteristic to a successful wildlife detection dog in a way that is suitable for the task of detecting leverets in their natural habitat (i.e. high work and play drive, high will to please, versatility and used for work in rough outdoor environments). However, other breeds or mixes can be suitable too. We decided for a puppy instead of an adult to make sure the dog would grow up in a controlled environment preparing it for the very delicate task of detecting live leverets. Using such an approach, chasing of any object can be prevented from very young age on. One male puppy was selected when it was nine weeks old. At the age of seven months, the puppy was evaluated for its suitability for detection work (reward drive (toy), work endurance, cooperation with handler) and subsequently entered the training phase. The dog was initially trained on residual scent, i.e. scent of a leveret but not an actual live leveret. Training aids are sources of target (residual) scent used to teach the dog the desired target that it is required to find and alert upon (by methods based on operant conditioning) (Cablk and Heaton, 2006). Training aids for this study were fleece toys rubbed on the back of a live leveret, cotton bags that were previously used to transport leverets after catching as well as dried leveret fur. Concerning live target detection, impulse control is a very important part of the training. We trained a freeze response to moving triggers, i.e. when dragging along a fur-dummy in front of the dog while searching for leverets it was required to freeze instead of chasing after it. When doing so the dog was rewarded with a toy (note that it was never allowed to get the fur-dummy as a reward). On the other hand, it was important to keep up the dog’s interest in wildlife so that in the end the dog would still be eager to search for leverets but once detected would not harm them. Furthermore, general obedience training was used to make sure the dog was controllable in every possible situation.

As soon as the dog was able to reliably find and indicate the training aid objects (ca. 5 months of training including impulse control, making sure the dog would not chase any moving objects), we introduced the dog to live leverets previously found by using thermal imaging. Such a setup allowed us full control of the dog and real target training. However, such ideal training conditions were rare (n=12) as the areas with known leveret locations were sparse, due to small hare population size in the study areas and in addition some locations were not suitable for controlled dog training (e.g. very thick vegetation). Furthermore, we limited exposure of individual leverets (n=9) to the dog’s training procedure to minimize disturbance. First contact with a live leveret occurred when the dog was one year old. Training sessions with live leverets (n=12) took place in February, June and August 2014. After these training sessions the dog was able to find and indicate live leverets (for which the

location was previously known) so that we could start using the dog for searches on unknown leverets. During the training phase, the dog had learned to walk on transect lines the handler indicated to him and thereby learned to systematically cover the search area. The fact that we did not have our target animal available for training ad libitum but only occasionally (n=12, spread over a 7-month period), prolonged the training period substantially resulting in the use of the detection dog only during the last 11 months of the study and only during 3 % of the total search time.

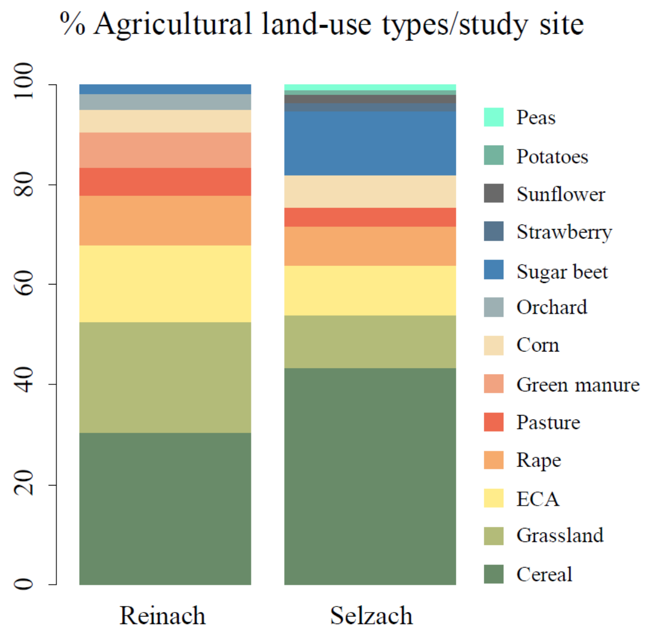

Fig. S1 Proportions of agricultural land-use types/study site (Reinach (BL) and Selzach (SO), Switzerland). Numbers of hectares (ha) per land-use type were averaged over the three study years (2013 – 2015) and subsequently divided by the total agricultural area, which was 103 ha for Reinach and 291 ha for Selzach. ECA = agricultural compensation areas, including extensively managed grassland, fallow land, edges and hedges.

Literature Cited

Cablk, M. E., & J. S. Heaton. (2006). Accuracy and Reliability of Dogs in Surveying for Desert Tortoise (*Gopherus Agassizii*). *Ecological Applications*, 5, 1926-35.

Karp, D. (2019). Preweaning behaviour and mortality in wild brown hare leverets (Lepus europaeus). PhD thesis, University of Zurich.
